# Properties of traditional bamboo carrying poles have implications for user interactions

**DOI:** 10.1371/journal.pone.0196208

**Published:** 2018-05-10

**Authors:** Ryan T. Schroeder, James L. Croft, Giang D. Ngo, John E. A. Bertram

**Affiliations:** 1 Biomedical Engineering Graduate Program, University of Calgary, Calgary, Alberta, Canada; 2 Centre of Exercise and Sports Research, School of Medical and Health Sciences, Edith Cowan University, Joondalup, Perth, Western Australia, Australia; 3 Agriculture and Forestry, Thai Nguyen University, Thai Nguyen, Vietnam; 4 Cumming School of Medicine, University of Calgary, Calgary, Alberta, Canada; University of Colorado Boulder, UNITED STATES

## Abstract

Compliant bamboo poles have long been used for load carriage in Asian cultures. Although this custom differs from Western conventions of rigid body attachments (e.g. backpack), potential benefits include reduced peak shoulder forces as well as metabolic transport cost savings. Evidence that carrying a flexible pole benefits locomotion remains mixed, perhaps in part because the properties of pole design (e.g. bamboo material, structural geometry, etc.) have largely been neglected. These properties influence vibrational forces and consequently, the energy required by the user to manage the oscillations. We collected authentic bamboo poles from northern Vietnam and characterized their design parameters. Four poles were extensively studied in the lab (load-deflection testing, resonance testing, and computed tomography scans of three-dimensional geometry), and 10 others were tested at a rural Vietnamese farm site (basic measures of form and resonance). A mass-spring-damper model was used to characterize a relationship between resonant frequency (which affects the energetics of the pole-carrier system) and pole properties concerning stiffness, damping, etc. Model predictions of resonant frequencies agreed well with empirical data. Although measured properties suggest the poles are not optimally designed to reduce peak oscillation forces, resonant frequencies are within range of a typical human walking cadence, and this is likely to have a consequence on locomotion energetics.

## Introduction

**Fig 1:**
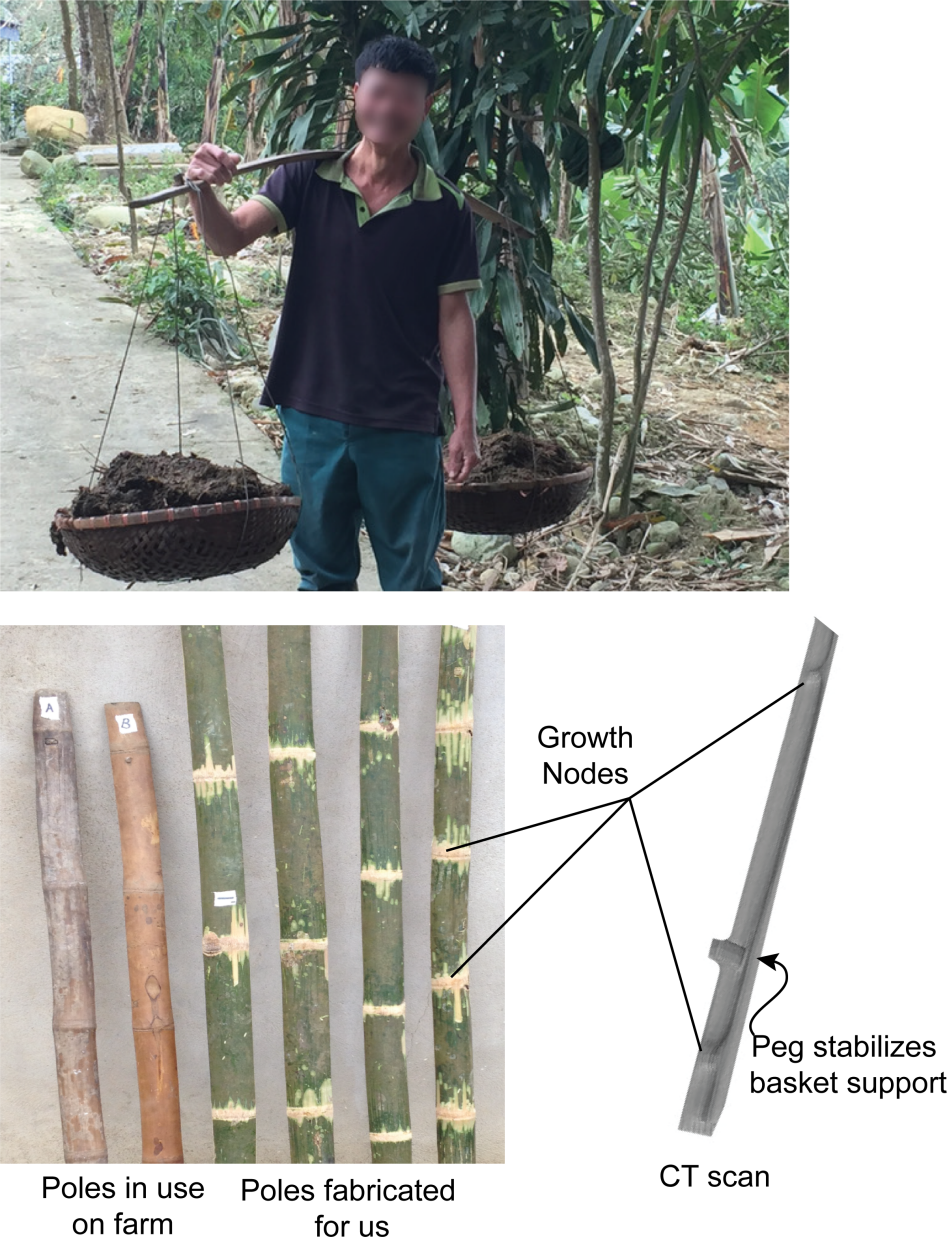
Pole carrying technique and example poles. Top: A farm worker carries a bamboo pole in northern Vietnam. The pole is supported at the shoulder with the hand (same side as the supporting shoulder) resting on top of the forward end to steady the system. Growth nodes are also indicated (Bottom Left: poles used in the study; Bottom Right: CT scan of pole C). These nodes are characterized by a thickening of the cross-section from a portion of a hollow tube to a portion of a solid cylinder.

Human load carriage remains an important part of working life in various cultures around the world, and this has led to the development of diverse carrying strategies. One notable example is the use of flexible bamboo poles in Southeast Asia. These resilient tools are typically placed on the shoulder to facilitate carrying of substantial loads (often as much as body weight or more) as well as awkward or bulky loads for farm work and transportation to the marketplace (Fig 1). This is of particular interest in locomotion research because the flexible pole may influence the metabolic expenditure required to transport loads. However, there is conflicting evidence supporting this hypothesis. Specifically, some researchers have found a slight increase in metabolic cost (+3%) whilst others have found a decrease (-5%) for carrying with a compliant pole [1,2]. Although it is not the focus of this paper we highlight these studies to show how material/structural properties of the pole may have an effect on locomotion energetics.

Kram described a dynamic interaction in which the pole’s deflection allows the load to travel in a relatively flat trajectory compared to the carrier’s body mass during locomotion, thus reducing the mechanical work (proportionate to the load’s oscillation amplitude) required to lift the load with each step [1]. He used polyvinyl chloride (i.e. PVC) pipes as a proxy for bamboo poles to explore whether general flexibility might have this effect on human subjects performing a running gait. Although the relatively low stiffness of the plastic poles (approximately 523 Nm^-1^ in Kram’s study) reduced peak forces acting on the shoulder, increases in metabolic expenditure (+22% for a 19%-of-body-weight load) were mostly in line with studies showing that metabolic cost increases approximately proportionately to the mass of a load carried with a backpack or waist harness [3,4]. In other words, the plastic poles used in Kram’s study did not save energy and metabolic cost was similar to the expected cost of carrying the load in a standard backpack, despite differences in stiffness and other influential parameters, such as damping.

In a subsequent study, Castillo et al. compared the metabolic cost of transport for carrying a total load of 170 N (17.3 kg) using rigid steel poles and bamboo poles (that they fabricated themselves) [2]. In contrast to the previous study, the cost was reduced by approximately 5% when using the bamboo pole compared to the steel pole over a defined range of walking step frequencies. The authors performed a basic vibration analysis to show that resonance (i.e. the fundamental oscillation frequency at which the flexible pole vibrates freely) can influence the energetics of locomotion. This is because the magnitude (and phase) of the vibrations—as well as reaction forces felt by the individual—occur as a function of oscillation frequency, and this is largely determined by the step frequency of the user [1,2,5–9].

More specifically, Castillo et al. implied that a carrier should walk with a step frequency slightly higher than the resonant frequency of the pole-load system in order to receive an energetic benefit. At this relative frequency, the pole and load oscillate at a relatively high magnitude while out of phase with the vertical body oscillation of the carrier. Ultimately, it is theorized that this interaction should require less leg work by the carrier, since the summed mass of the system stays relatively flat (the load is low when the body is high and vice versa, where motion cancels when summed). The apparent contradiction in results of studies by Kram and Castillo et al. may be due to a variety of factors (e.g. walking versus running gaits, pain or discomfort carrying with a steel pole, etc.). However, the type of pole (e.g. material, structure, etc.) and, consequently, its properties can likely have an important influence on the energetics of load carriage.

Potwar et al. [10] recognized the importance of pole properties in a study that described a design parameter optimization model constraining stiffness, weight of the pole, and strength (in order to mitigate mechanical failure). The explicit intent of this model was to identify a range of pole dimensions minimizing peak forces on the shoulder for both walking and running gaits. Although this theoretical analysis successfully determined optimal design parameters for load carrying, the structural and material properties of authentic bamboo poles (i.e. fashioned by individuals using them daily) have not been rigorously evaluated within the context of locomotion energetics and load carrying.

The purpose of this study was to characterize the design parameters of authentic bamboo poles used in traditional load carrying by Vietnamese farmworkers. Although multiple considerations are likely to influence the fabrication of a carrying pole, two specific design outcomes were evaluated: reduction of both (1) peak forces to the body and (2) energetic expenditure of the carrier. The former was evaluated by comparing pole properties in this study to those determined as optimal by Potwar et al. [10]. The latter was evaluated by comparing resonant frequencies measured in this study to those associated with a reduced metabolic cost of the carrier [2].

To accomplish this analysis, we performed testing in rural northern Vietnam (farm site) as well as in the lab. Conditions at the farm site meant we were only able to make simple evaluations (10 poles). However, four additional poles were fabricated by a local craftsperson at the farm site with local materials, and these were subsequently brought back to the lab for more thorough evaluation. The data from the lab-tested poles (LPs) were used to determine detailed mechanical and structural properties and validate a theoretical model describing dynamic pole behaviors. This model was then used to determine the same set of properties and design parameters—albeit indirectly, through the model’s outputs—for the 10 farm-tested poles (FPs).

Specifically, Euler-Bernoulli equations (i.e. classical beam theory) were used to characterize stiffness of a mass-spring-damper system describing load oscillations. The purpose of this model was to characterize a relationship between resonance behaviors and fundamental properties of the bamboo poles in order to assess potential influence on human locomotion. Insights from this study should prove useful to the understanding of load carriage with a flexible apparatus. In particular, the implications of design strategies on reaction forces and energetics are discussed. While many potential benefits have previously been identified for the implementation of such devices, authentic bamboo poles fabricated with traditional techniques have not been evaluated. Design attributes are inferred from empirical and theoretical analysis described further in the following sections.

## Methods

Two experiments (resonance and load-deflection) were performed in order to test relevant mechanical and structural properties. These data were used in a theoretical model describing the relationship of resonance and other dynamic behaviors. Furthermore, computed tomography (CT) scans were used to image the LPs and measure basic geometric parameters associated with cross-sectional profiles along the length of each pole.

**Fig 2:**
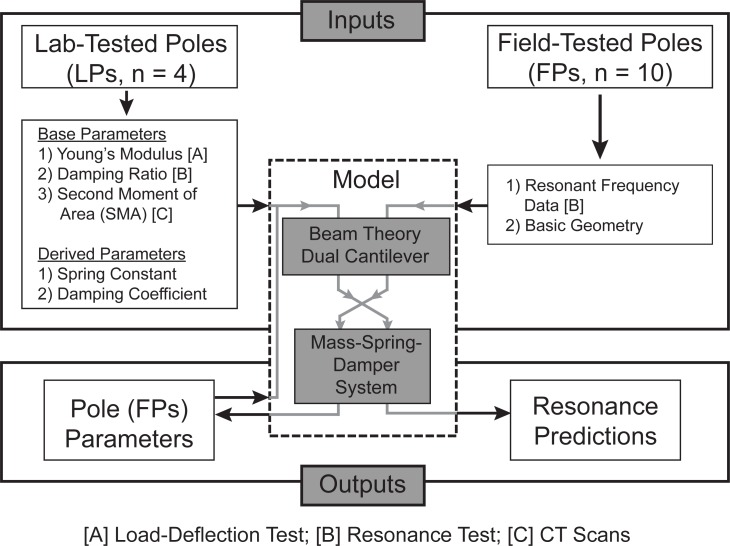
Methods flowchart. The flowchart indicates experimental data/results used in the model to quantify pole parameters and resonance predictions for the lab- and field-tested poles. Note: properties labeled with [A], [B], or [C] were determined from the corresponding test indicated at the bottom of the flowchart.

The physical properties explored in this study can be grouped into two categories: (1) *base* and (2) *derived*. The *base* group comprises Young’s modulus (i.e. *E*, modulus of elasticity), hysteresis (*hys*), damping ratio (*ζ*), basic geometric parameters, and second moment of area (*I*). The *derived* group includes spring constant (*k*) and damping coefficient (*c*). A flowchart describing testing and analysis of the two pole groups is shown in Fig 2.

### Resonance test

In the first test, resonant frequencies were measured for the poles oscillating during free vibration over a range of loads. To accomplish this, a rigid testing frame was constructed. Two aluminum I-beams (S 3 in X 7.5 in, ASTM A6) were clamped across a steel frame solidly connected to both the floor and ceiling in a reinforced concrete building. Each pole was tightly clamped at a single attachment point at its functional center, which was determined by balancing the pole on the shoulder (with an arbitrary but equal load at each end of the pole). The functional center often did not coincide with the geometric center of the pole due to variance in density as well as an extra moment created by the weight of the carrier’s hand laying over the top of the pole, in the natural carrying style used by the indigenous Vietnamese farmworkers (see Fig 1). Because of this imbalance, functional centers tended to lie closer to the front of each pole. The functional center was chosen in order to more closely replicate pole loading as it would be seen in practice.

When mounted to the frame, two limit stops (wooden pegs) were placed just above the neutral height of each end of the pole (i.e. the height of the pole ends while under no load). This served to ensure that the resonant frequencies only characterized the stiffness of the pole bending in its functional direction—downward. Next, the testing pole was loaded with lead weights (21.82–201.09 N or 2.225–20.505 kg applied equally to each end of the pole, in intervals of 21.82 N or 2.225 kg). Baskets and wire supports (commonly used and purchased in Vietnam) were used to cradle the weights (Fig 1) and added an additional 4.71 N (0.480 kg) to each end. With each loading level, the pole ends were held up by hand until they lightly touched the limit stops (neutral position). Data collection was synchronized to the release of the pole from its neutral position. The loaded pole was allowed to oscillate under free vibration for 30 seconds (this duration was adequate to allow all poles to come to rest under any of the applied loads). Throughout all tests, the pole ends did not touch the limit stops, thus ensuring the correct direction of bending.

Inertial sensors (Xsens MTw, Xsens Technologies, Enschede, The Netherlands) were used to measure the vertical acceleration of the loads during free vibration (one at each end of the pole) and displacement was subsequently calculated by double integration with initial conditions. Mean values and standard deviations of the oscillation frequency were calculated for all poles and under a range of loading. Furthermore, the decay of the displacement signal was determined via:

$$\varphi =\ln\left(\frac{y_{i}}{y_{i+1}}\right),y_{i}>y_{i+1}$$ (1)

where *φ* is the logarithmic decrement; *y*_*i*_ and *y*_*i+1*_ are the magnitudes of two consecutive signal peaks. The logarithmic decrement was noted for each cycle until the signal decayed completely and the median value was chosen to characterize the signal. This median value was then used in Eq (2) to calculate the damping ratio—signal decay relative to a critically damped system.

$$\zeta =\frac{\varphi }{\sqrt{{\left(2\pi \right)}^{2}+\varphi^{2}}}$$ (2)

### Load-deflection test

Stiffness and hysteresis properties of the poles were characterized using a load-deflection test. Each pole was fixed to the rigid frame at its functional center. High contrast markers were placed along the length of each pole (at each growth node and intermediate between each node, see Fig 1). A total of 12–13 points were measured, depending on the number of nodes per pole.

A digital camera (Casio EX-ZR700) was placed perpendicular to the pole at a distance of 10 m (to minimize parallax and lens distortions). Continuous video (30 Hz) recorded pole deflection under a series of loads placed in the baskets attached at each end of the pole. Starting from a zero load position, successive weights of 21.82 N (2.225 kg) were added to both baskets until a total of 201.09 N (20.505 kg) was applied (nine weights in each basket, overall pole load 402.17 N or 41.010 kg). The pole was allowed to settle to a constant deflection following the addition of each weight. These weights were then removed in succession so the pole’s relaxation could be recorded. The images were calibrated, and a marker on the frame was used to verify that support frame deflection was negligible. For all test videos, the support frame’s deflection was measured as less than a pixel, and thus, the frame was considered to be ideally fixed and rigid. All marker videos were digitized in MATLAB (The MathWorks Inc., Natick, Massachusetts) using the DLTdv5 software program [11].

Displacements were determined by subtracting the initial (i.e. zero-load) positions from the deflection positions for each load. All positions were measured by averaging the data over each ten-second interval (after any basket sway was brought to rest). The standard deviation of each position was also determined for each load increment.

Load-deflection curves were used to depict deflection at the load attachment peg for a full cycle of loading and unloading. The area between the curves was calculated in order to quantify strain energy lost to hysteresis, defined as:

$$hys=\frac{\int_{0}^{\delta_{max}}F^{+}(\delta )d\delta -\int_{0}^{\delta_{max}}F^{-}(\delta )d\delta }{\int_{0}^{\delta_{max}}F^{+}(\delta )d\delta }$$ (3)

where *δ* is deflection, *F*^*+*^ is the curve for loading and *F*^*-*^ is the curve for unloading. The concept of resilience as strain energy returned by the system can also be defined as *res* = 1 − *hys*.

### Model

Simple beam theory was used to determine the Young’s modulus of the bamboo. Specifically, two cantilever beams were considered—one for each end of the pole—with a single concentrated load applied at the load attachment peg. Note this model assumes that no net translational or rotational motion should occur about the contact point at the carrier’s shoulder. Although this assumption is likely violated in practice, experienced users typically maintain a balance of forces at the shoulder (a technique facilitated by the hand resting on the front end of the pole, see Fig 1) for increased system stability.

In order to assess pole compliance, a deflection surface was mapped over two parameters: distance from the fixed functional center to each marker along the pole and weight of the load. A least squares non-linear regression was fit to this surface via the following model, derived from simple beam theory for a cantilever beam:

$$\delta =a(3mgx_{L}x^{2}-mgx^{3}),a=\frac{1}{6EI}$$ (4)

where *δ* is the deflection, *m* is mass of the load, *g* is gravitational acceleration (9.81 ms^-1^), *x* is the distance from the fixed functional center of the pole to a given marker, *x*_*L*_ is the distance from the fixed functional center of the pole to the load attachment point, *E* is the Young’s modulus of the bamboo and *I* is the second moment of area of the pole’s cross-section at the marker of interest. From this model, the flexural rigidity (E*I) was determined for each pole.

Next, a mass-spring-damper model was used to determine a theoretical relationship for the damped resonant frequency of the system. The equation of motion for this one degree of freedom system is:

$$m\ddot{y}+c\dot{y}+ky=mg$$ (5)

where *y* describes motion of the load along a vertical axis (positive is defined downward – the assumed direction of the pole’s deflection under load), *c* is the damping coefficient, equivalent to the expression *2ζω*_*n*_*m* where *ω*_*n*_ is the natural frequency of the oscillating system during free vibration when no damping is present and *k* is the spring constant describing the relationship between force and deflection. A cantilever beam model was used to show this relationship in Eq (4). Here however, the spring constant influencing the load is only relevant for the case when *x = x*_*L*_. Also, since deflection is equivalent to the displacement of the load, *δ* is substituted with the spatial variable *y*. A further adaptation allows for a dynamic point load, *P(y)*, that can change as a function of the load’s displacement. Note that in the static form, *P* is simply the weight of the load, as in Eq (4). After these adjustments, the spring constant can be defined as:

$$P=\frac{3EI}{x_{L}^{3}}y=ky$$ (6)

With the mass-spring-damper system described, a damped resonant frequency (*ω*_*DR*_) is calculated from the expression in Eq (7), where $\frac{3EI}{x_{L}^{3}}$ is substituted for *k* via Eq (6):

$$\begin{array}{l} \omega_{DR}=\sqrt{1-\zeta^{2}}*\sqrt{\frac{k}{m}}\\ \;=\sqrt{1-\zeta^{2}}*\sqrt{\frac{3EI}{mx_{L}^{3}}} \end{array}$$ (7)

Eq (7) provides a theoretical prediction of the pole-load’s damped resonant frequency via a mass-spring-damper system and simple beam theory. This model was used to compare the frequencies measured in the resonance test with the theoretical frequencies predicted by basic pole properties measured directly in the load-deflection test.

### CT scans and geometric model

Three-dimensional images of the bamboo LPs were acquired using computed tomography (GE Revolution GSI, General Electric, Milwaukee, WI, USA). Scan parameters were selected (120 kVp, 99 mA, pitch 1:1) to produce images with a voxel size of 0.625 mm x 0.625 mm x 5 mm (width x height x length; see S1 STL, S2 STL, S3 STL, and S4 STL for respective CT data as STL files). Slices of the images were analyzed at 5 mm intervals along the longitudinal axis of each pole. This analysis included a determination of width, height, cross-sectional area, centroid, and second moment of area for each slice. To calculate these parameters, linear interpolation was used to consider the culmination of vertices as a polygon in a given slice. Because the resolution of the scanner is sufficiently high, errors introduced by the linear interpolation are negligible. The calculations for centroid, area, and second moment of area for a polygon are shown in Eqs (8–10) [12]:

$$\begin{array}{l} C_{z}=\frac{1}{6A}\sum_{i=0}^{n-1}\left(z_{i}+z_{i+1}\right)\left(z_{i}y_{i+1}-z_{i+1}y_{i}\right)\\ C_{y}=\frac{1}{6A}\sum_{i=0}^{n-1}\left(y_{i}+y_{i+1}\right)\left(z_{i}y_{i+1}-z_{i+1}y_{i}\right)\\ C=\left(C_{z},C_{y}\right) \end{array}$$ (8)

where *C* is the centroid of the shape, *z* and *y* are the horizontal and vertical components of the coordinate system, respectively, *n* is the total number of vertices in a cross-sectional slice and *i* represents a particular vertex being processed by the algorithm. Also, *A* is the area of the defined shape [12]:

$$A=\frac{1}{2}\sum_{i=0}^{n-1}\left(z_{i}y_{i+1}-z_{i+1}y_{i}\right)$$ (9)

Further, the second moment of area was determined for the polygon-shaped section of each CT slice using the following algorithm [12]:

$$I_{zz}=\frac{1}{12}\sum_{i=0}^{n-1}\left(y_{i}^{2}+y_{i}y_{i+1}+y_{i+1}^{2}\right)(z_{i}y_{i+1}-z_{i+1}y_{i})$$ (10)

**Fig 3:**
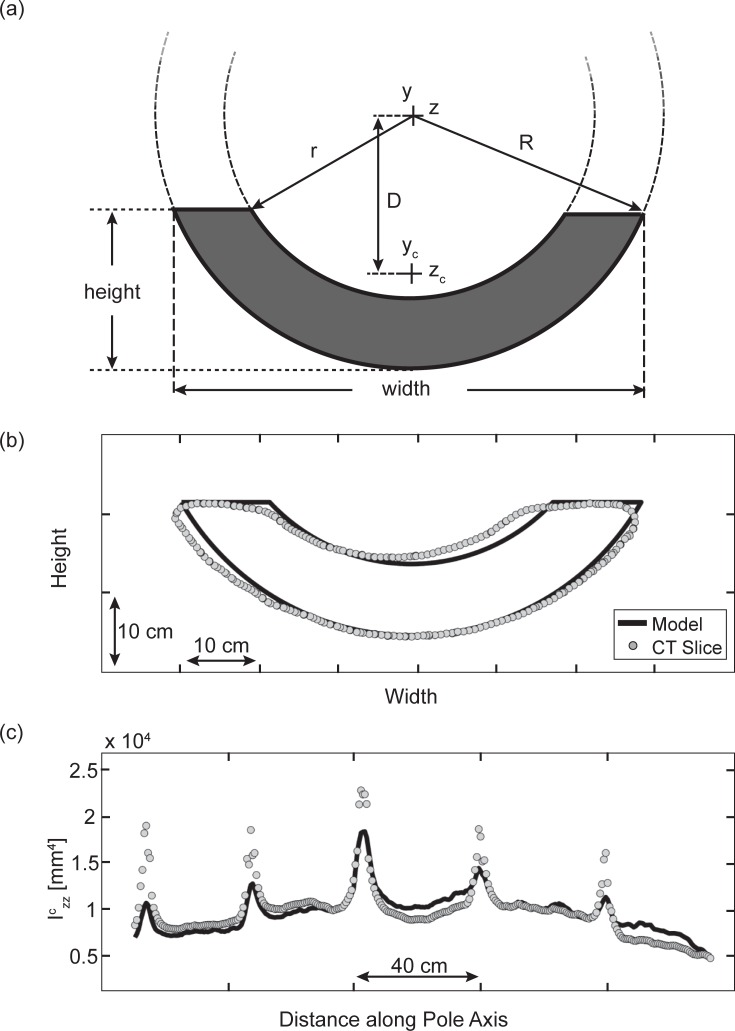
Geometric data and model outputs. (a) The pole model geometry is shown in the shaded portion at the bottom of a tubular cross-section. (b) An example slice from a CT scan of Pole C (LP) is shown. (c) The second moment of area for all slices of the example Pole C) are plotted. Gray circles indicate empirical data and solid black lines are the model’s outputs.

Mean values of the second moment of area were recorded for all LP measurements within the center region bordered by the nearest growth nodes (bamboo grows to form a hollow stem that is fairly uniform between horizontally thickened nodes). The FPs were measured (by hand) at the functional center only, and a geometric model was used to approximate the second moment of area along the length of the pole. This model is essentially a horizontal portion of a tubular cross-section (see Fig 3A) and requires two simple parameters as inputs: (i) height (while the pole lays flat) and (ii) width. The outer radius *R* and other important parameters were calculated from the input values. Since the inner radius *r* is not available from this model, it was scaled in direct proportion to the outer radius. This proportionality constant ranged from 0.69 to 0.78 for a variety of poles and a mean value 0.73 was used as an approximation in the model.

The second moment of area of the geometric model (see Fig 3A) was calculated for the FPs by subtracting the inner circle from the outer circle. Horizontal elements were integrated over the vertical range (i.e. height) of the shape relative to a coordinate system located at the center of the concentric circles.

$$I_{zz}=\int y^{2}\;dA=2\int_{-R}^{-R+h}(y^{2}\sqrt{R^{2}-y^{2}})\;dy-2\int_{-R}^{-R+h}(y^{2}\sqrt{r^{2}-y^{2}})\;dy$$ (11)

where *I*_*zz*_ is the second moment of area about the horizontal axis passing through the center of the concentric circles, *y* is the vertical coordinate relative to this center, *R* is the outer radius, *r* is the inner radius, and *h* is the height of the tubular portion. The parallel axis theorem was used to determine the second moment of area relative to the centroid of the cross-sectional shape.

$$I_{zz}^{c}=I_{zz}-AD^{2}$$ (12)

where $I_{zz}^{c}$ is the second moment of area relative to the center of the cross-sectional shape, *A* is the area of the shape and *D* is the distance from the center of the concentric circles to the centroid of the tubular portion. Eq (12) was used to calculate the second moment of area for both LPs and FPs.

## Results

### Stiffness and hysteresis

**Fig 4:**
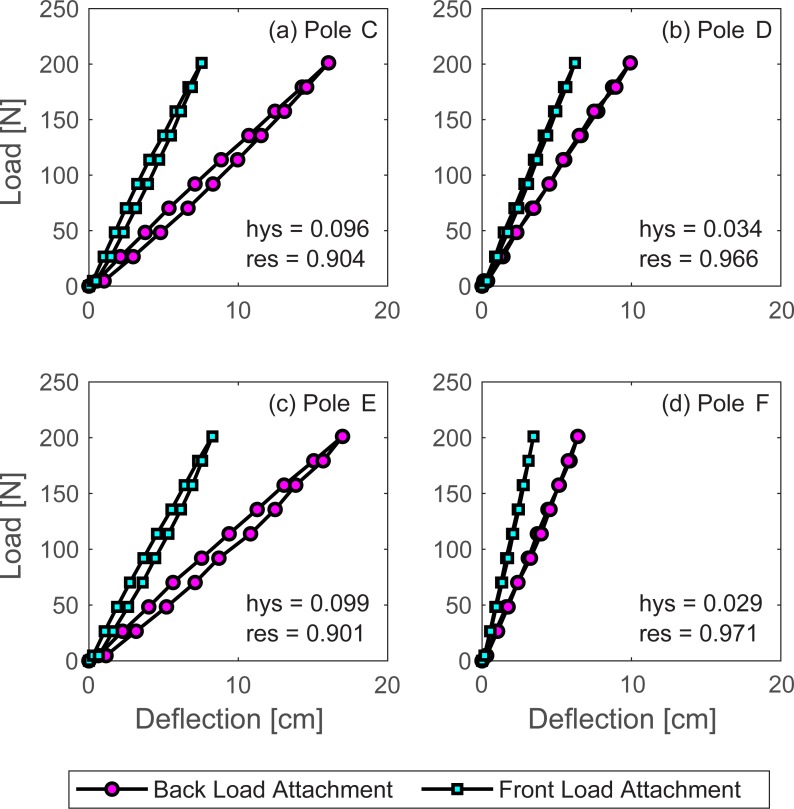
Load-deflection curves for the LPs. A full cycle of loading and unloading is shown for each end of each LP: (a) Pole C, (b) Pole D, (c) Pole E, (d) Pole F. Note that standard deviation of all deflection measurements are well below ±0.01 cm and cannot be viewed in these plots.

The stiffness of a linear system is commonly characterized by the slope of its load-deflection curve where a steeper slope implies a structurally stiffer system. These curves describe the deformation at the load attachment point for a full cycle of loading/unloading and are shown for each of the LPs (see Fig 4). It should be noted that the different slopes in each pair of curves is primarily due to a functional center that is biased towards the front of the pole. As a result, the curves representing the front end of each pole (shorter length) tend to be stiffer. The average hysteresis [see Eq (3); Fig 4] and resilience are also listed for each pole. Hysteresis values ranged from 2.9% in Pole F to 9.9% in Pole E. These values indicate relatively modest energy losses due to damping.

### Young’s modulus

**Fig 5:**
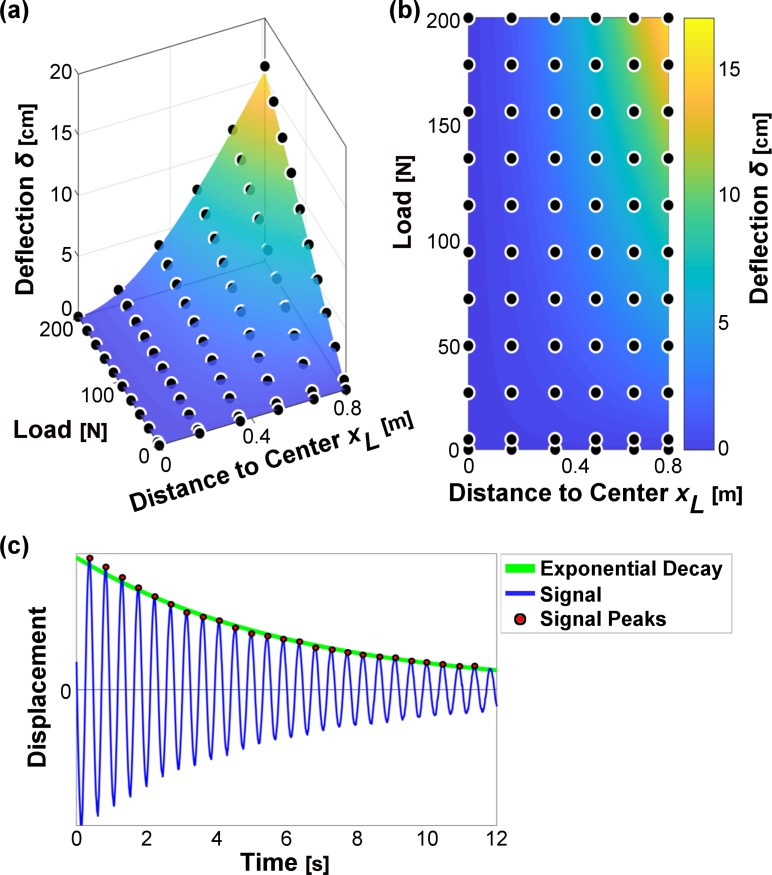
Examples of load-deflection surface and resonance testing data. (a) An example of the least-squares non-linear regression model fit to data from Pole C (LP) is shown. Note that the surface is linear with respect to load and nonlinear with respect to distance of the loading point from the center. (b) The contour map shows the curvature of the surface with yellow shades indicating more deflection and blue shades less deflection. (c) An example of signal decay for a pole and load under free vibration is shown. The thick green line follows the exponential decay of peak signal magnitude while the thin blue line shows the vertical oscillations measured with inertial sensors placed at the load attachment points. Note that scaling of the vertical plot axis is not labeled since absolute magnitude is irrelevant for this test.

Although the plots shown in Fig 4 illustrate pole stiffness over a range of loads, each pair of curves only indicates deflection for two discrete points at the basket attachment points near the ends of the pole. However, multiple points were measured along each pole’s axis during the stiffness test. Thus, in order to more thoroughly characterize stiffness of the LPs, a surface was plotted where the vertical axis indicates deflection and the horizontal axes are load and distance (from the fixed center to the point of deformation along the pole’s axis). A least-squares non-linear regression was used to fit the data to a theoretical surface derived from classical beam theory for a cantilever beam [see Eq (4); Fig 5A and 5B]. The coefficient *a*—defined in Eq (4)—was determined from these regressions in order to solve for the Young’s modulus, *E*, of the bamboo material.

The resulting regression coefficients, *a*, are reported for poles labeled *C-F* (LPs) as best estimate (95% confidence intervals) (x10^-4^ s^2^kg^-1^m^-3^): 7.29 (7.25–7.34), 5.14 (5.12–5.16), 8.56 (8.53–8.60) and 3.98 (3.94-4.02), respectively. Upon measuring the second moment of area (see section 3.4) the Young’s Moduli were calculated from the best fit regression coefficient and ranged from 14.7-22.2 GPa for the LPs. Furthermore, the spring constant, *k*, was determined for the load acting at the basket attachment point on the pole via the relationship given in Eq (6). The resulting values ranged from 1.31–3.59 kNm^-1^ for the LPs.

The spring constant was also determined for the FPs. However, instead of calculating this parameter from its relationship to Young’s modulus, it was determined from the coefficient of a least-squares non-linear regression applied to resonance test data. The non-linear model used for this regression is given by Eq (7). The spring constant values determined from this regression ranged from 1.83-4.18 kNm^-1^ for the FPs. After calculating the second moment of area, the Young’s modulus was determined for all of the FPs using the best estimate of the spring constant. These values ranged from 10.1-21.0 GPa for the FPs.

### Damping ratio and damping coefficient

The damping ratio was calculated from resonance behavior by characterizing the exponential decay of signal peaks over time (see Fig 5C). Damping ratio results ranged in value from 0.010-0.013 for the LPs and 0.011-0.018 for the FPs. The average damping coefficients were calculated from these results: 2.77-3.56 Nsm^-1^ for the LPs and 3.45-7.28 Nsm^-1^ for the FPs.

### Second moment of area

Fig 3B and 3C show a comparison of the dimensional measurements of an example pole (LP-C) from the CT scan with the corresponding model geometry. The middle panel (*b*) shows an overlay of the model and the CT scan while the bottom panel (*c*) shows the second moment of area data calculated from both the model and the CT scan for all slices of Pole C. Although there are subtle differences between the scans and the model, it gives a reasonable representation of the pole geometry.

Although the second moment of area tapers slightly toward the ends of the pole (the form cut by the Vietnamese craftsperson who fabricated each pole), these systematic trends are modest, confirming that the cross-sectional geometry is relatively consistent along the pole’s axis. More prominent fluctuations are found at fairly regular intervals where the second moment of area spikes. These spikes occur at the pole’s growth nodes, however their influence on the deflection of the structure is likely modest given their small contribution to the total length—essentially brief interruptions to an otherwise consistent cross-section. Mean values for geometric parameters—determined from the middle section bordered by the nearest bamboo nodes—were used to characterize the entire pole. The widths of the poles range from 55.7-61.4 mm for the LPs (measured with the CT scans) and 48.0-62.0 mm for the FPs (measured by hand at the farm site). The heights of the poles range from 17.9-25.0 mm for the LPs (CT scans) and 18.0-24.0 mm for the FPs (hand measurements). Finally, the second moment of area measurements are reported as follows: 1.028-2.740x10^4^ mm^4^ for the LPs and 1.078-2.254x10^4^ mm^4^ for the FPs.

### Model predictions and empirical resonant frequency

**Fig 6:**
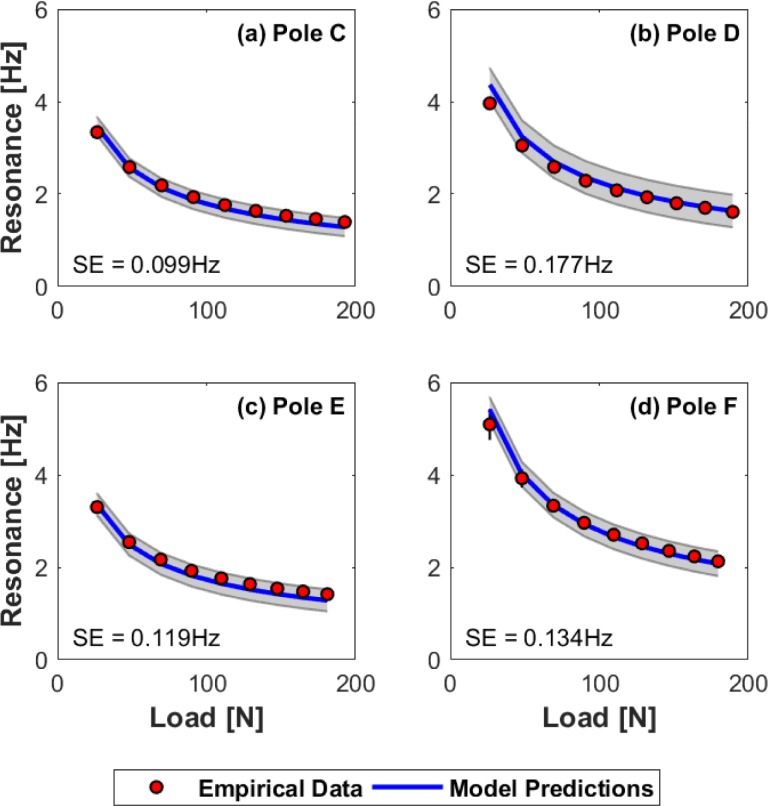
Resonant frequency curves for the LPs. The relationship between resonant frequency and load is shown for each of the LPs. Circles are mean frequencies measured empirically during free vibration, and the solid line indicates resonant frequencies predicted by the mass-spring-damper model. Two standard errors of each model approximates 95% confidence intervals and is indicated by the gray shaded region. Standard deviations of the empirical means are also shown by the error bars of individual data points. Note, much of this error is too small to be visible at the scale of these plots. (a) Pole C (b) Pole D (c) Pole E (d) Pole F.

The predictive capacity of the mass-spring-damper model was assessed by comparing it to empirical data of free vibration under various loads. Resonant frequencies associated with the lowest load were approximately 3–5 Hz while frequencies at the highest load were approximately 1–2 Hz. Standard error (SE) of the model ranged from ±0.099-0.177 Hz (or 5.11–7.54% of the frequency range over all tested loads) for the LPs (see Fig 6).

**Fig 7:**
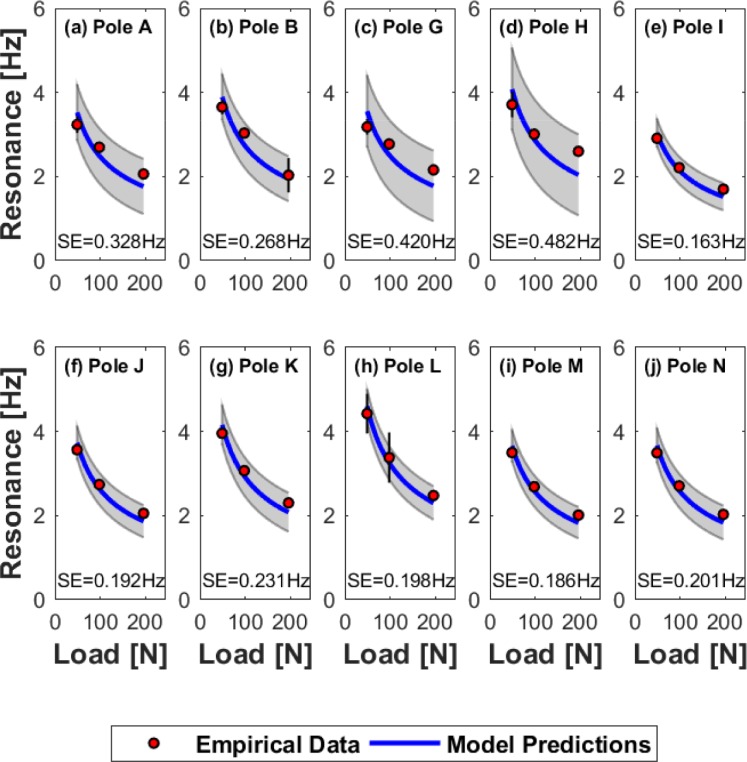
Resonant frequency curves for the FPs. The relationship between resonant frequency and load is shown for each of the FPs. Circles are mean frequencies measured empirically during free vibration, and the solid line indicates resonant frequencies predicted by the mass-spring-damper model. Two standard errors of each model approximates 95% confidence intervals and is indicated by the gray shaded region. Standard deviations of the empirical means are also shown by the error bars of individual data points. Note, much of this error is too small to be visible at the scale of these plots. (a) Pole A, (b) Pole B, (c) Pole G, (d) Pole H, (e) Pole I, (f) Pole J, (g) Pole K, (h) Pole L, (i) Pole M, (j) Pole N.

Model predictions were also compared to data gathered in the field (FPs) where the standard error ranged from ±0.163–0.482 Hz (or 8.61–23.64% of the frequency range over all tested loads). For this sample, resonant frequencies for the lowest load were approximately 3.0–5.0 Hz while frequencies at the highest load were approximately 1.5–2.5 Hz (see Fig 7).

### Summary of pole properties

One important function of the model is as a tool to predict fundamental pole properties without explicit measurements. Thorough assessment and characterization of the four LPs verified the resonance predictions of the model. Assuming that the LPs are a representative sample of the larger bamboo pole population, properties of the FPs were also estimated from the model. The results of these properties are summarized in Tables 1 and 2, as well as the properties that were measured directly for the LPs.

**Table 1:** Summary of pole geometry and inertia.

Testing	Pole	Mass [kg]	Second Moment of Area [10^4^ mm^4^] mean (±SD)	Width [mm] mean (±SD)	Height [mm] mean (±SD)	Outer Radius [mm]	Length [m]
lab	C	0.70	1.028 (0.059)	61.4 (0.8)	17.9 (0.5)	35.3	1.550
lab	D	0.83	1.538 (0.058)	60.6 (0.6)	22.5 (0.3)	31.7	1.573
lab	E	0.73	1.322 (0.052)	58.2 (0.2)	19.4 (0.2)	31.5	1.527
lab	F	0.98	2.740 (0.117)	55.7 (0.6)	25.0 (0.7)	28.0	1.409
field	A	0.85	1.862	60.0	22.0	31.5	1.297
field	B	0.85	1.422	59.0	20.0	31.8	1.256
field	G	0.90	1.191	62.0	18.0	35.7	1.237
field	H	0.94	1.689	61.0	21.0	32.7	1.272
field	I	0.94	1.650	60.0	21.0	31.9	1.424
field	J	0.76	1.078	48.0	20.0	24.4	1.255
field	K	0.81	1.905	61.0	22.0	32.1	1.305
field	L	1.00	2.254	58.0	24.0	29.5	1.348
field	M	0.90	1.698	56.0	22.0	28.8	1.395
field	N	0.93	1.318	61.0	19.0	34.0	1.296

Values of inertial and geometric properties are listed for both the lab- and field-tested poles. Note that standard deviation (SD) is listed for some properties of the lab poles but not for the field poles. This is due to the nature of the measurements made (basic hand measurements for the latter).

**Table 2:** Summary of pole properties.

Testing	Pole	Spring Constant [kNm^-1^] (95% CI)	Young's Modulus [GPa]	Damping Coefficient [Nsm^-1^]	Damping Ratio median (±SD)	Hysteresis [%]
lab	C	1.47	22.2	2.77	0.011 (0.019)	9.4
lab	D	2.00	21.1	2.77	0.010 (0.016)	3.2
lab	E	1.31	14.7	3.41	0.013 (0.032)	9.6
lab	F	3.59	15.3	3.56	0.010 (0.022)	2.9
field	A	2.46 (1.20–4.16)	11.2	3.92	0.012 (0.012)	…….
field	B	2.99 (1.80–4.48)	10.1	4.24	0.012 (0.010)	…….
field	G	2.50 (0.95–4.78)	16.5	3.45	0.011 (0.034)	…….
field	H	3.28 (1.25–6.29)	16.7	5.51	0.014 (0.020)	…….
field	I	1.83 (1.25–2.52)	13.3	4.02	0.015 (0.015)	…….
field	J	2.75 (1.91–3.75)	21.0	5.23	0.015 (0.011)	…….
field	K	3.42 (2.29–4.76)	16.6	4.63	0.012 (0.010)	…….
field	L	4.18 (3.09–5.44)	18.9	7.28	0.018 (0.042)	…….
field	M	2.65 (1.84–3.59)	17.6	3.68	0.011 (0.010)	…….
field	N	2.66 (1.80–3.69)	18.3	4.34	0.013 (0.040)	…….

Values for stiffness and damping parameters are listed for both the lab- and field-tested poles. Note that 95% confidence intervals (CI) are listed for spring constant of the field poles but not for the lab poles, since varying methods of analyses were used for each sample. Hysteresis values are not reported for the field poles since this test was not conducted at the farm site.

The average Young’s modulus (mean±SD) was 18.3±3.9 GPa for the lab tested poles (LPs) and 16.8±2.6 GPa for the field tested poles (FPs), an 8.1% difference. Comparisons of the average spring constant of the LPs and the FPs are as follows: 2.09±1.04 and 2.87±0.64 kNm^-1^ a difference of 37.1%. Damping ratios were 0.011±0.001 for the LPs and 0.013±0.002 for the FPs, a difference of 20.2%. Damping coefficient results were 3.13±0.42 and 4.63±1.13 Nsm^-1^, 48.1% different. Finally, the second moment of area for the LPs and the FPs were 1.66±0.75 and 1.61±0.36 x10^4^ mm^4^, a 3.0% difference.

## Discussion

Despite the different methods used to determine properties of LP and FP samples (direct testing versus inference from the model), the mean values of the two groups are comparable. Damping coefficient differs the most between the two groups (48.1%). As damping coefficient was not measured directly, this is likely due to differences between these poles and the assumptions made in the model. Furthermore, damping coefficient is calculated from both damping ratio and spring constant–each of which contribute their own sources of variance and error. Damping ratio differs by 20.2% between the groups, however the absolute values are all very low (0.010–0.018). That is, this variance is largely irrelevant (from a dynamics standpoint) given that the lowest and the highest values still suggest the poles are quite resilient. There is also a difference between the mean spring constants of both groups (37.1%), though this difference is less than a standard deviation of the LP sample. Variance in the poles’ spring constants can be attributed to a number of factors including cross-sectional geometry, Young’s modulus of the bamboo, and pole length. In particular, the LPs tended to be longer than the FPs on average, contributing to lower spring constants even as Young’s moduli were mostly similar. The testing location may have also influenced some of the properties (e.g. Young’s modulus, mass, etc.). In particular, the FPs were tested in the humid, subtropical climate of northern Vietnam while the LPs were tested indoors in the relatively dry and moderate climate of Calgary, Alberta. The effects of acclimatization were monitored every two weeks for a three-month period after the LPs were first brought to the lab. During this time, only one property value changed meaningfully; the average mass of the LPs dropped from 0.90 to 0.81 kg. This compares to an average mass of 0.89 kg for the FPs (measured in Vietnam). It seems likely that this loss of mass can be attributed to a decreased moisture content associated with the drier testing climate of the LPs. It is likely that this contrast in moisture content may help to explain differences in damping properties of the two pole types (recall, the FPs had a 48.1% higher damping coefficient on average when compared to the LPs).

Regardless of the variation in properties and parameters between the two groups of poles, the average values tend to agree with previous literature describing bamboo properties. For example, the average Young’s modulus of all the poles (17.3 GPa) is consistent with values published in various studies: Lakkad and Patel [13] measured the Young’s modulus of bamboo (Kao Zhu and Mao Zhu species) in the orientation of individual fibers as 20.6 GPa; Amada and Lakes [14] tested multiple samples of bamboo (species not specified) with different moisture content and found a Young’s modulus for transverse bending ranging from 7.31–14.80 GPa. In the same study, loss tangent values indicated extremely low levels of damping (tan δ ≈ 0.01). However, the loss tangent values were slightly increased when the bamboo samples were subjected to thorough wetting (up to tan δ ≈ 0.015 after wetting). These findings align with the results of our study showing generally very low values for damping ratio and hysteresis, however slightly more damping with the humid FPs compared to the relatively dry LPs.

Although measured low levels of damping are consistent with previous studies, one limitation remains a lack of knowledge about the specific mechanism(s) for energy loss (e.g. viscous, structural, etc.). We chose viscous damping for our model primarily due to its extensive consideration in previous literature—for engineering and biological structures and materials [15–18]—as well as its ability to predict energy losses driven by various physical mechanisms (including viscous and non-viscous mechanisms). In many systems, different models for energy loss are relatively insensitive to the effect they have on the ultimate output of the model: in our case, resonance. Although a discussion of energy loss mechanisms is important, we opted for a more pragmatic approach to our modeling: namely, to predict the most dominant influences on system resonance.

Another limitation of the model involves the assumption of a constant cross-sectional geometry. Clearly, the CT data show that this is not precisely the case (see Fig 3C). However, cross-sectional fluctuations are modest when considering trends over the length of the pole (tapering at the ends) and abbreviated when considering localized inconsistencies such as thickening at the nodes. Thus, we argue that introducing model complications to incorporate these variations are unlikely to be worth the refined accuracy. Perhaps the most obvious approach to further evaluation is a finite element model derived from the CT scans. We rejected this approach because we felt it was not necessary for predicting fundamental resonance of the pole-load system. Furthermore, analysis of the FPs would not benefit from such a model, since CT scanners were not feasible on the farm site. Nonetheless, a future study looking to test our simple model and understand nuanced behaviors of the structure could certainly benefit from the finite element method.

In order to consider design parameters of the bamboo poles, their properties (either measured or calculated) are compared with optimized values suggested by the peak force minimization model developed by Potwar et al. [10]. The model predicted shoulder forces based on a spring loaded inverted pendulum locomotion model interacting with a beam-like pole (similar to the current study). They also took multiple constraints into consideration. A pole mass constraint was used to limit the mass of the pole to less than 10% of the total load, assuming published values of bamboo density and calculations of pole volume. A strength constraint was also applied by considering the theoretical mechanical stress required for failure (e.g. plastic deformation). A load clearance constraint was enforced by limiting pole stiffness (lower bound) to allow for a maximum of 0.4 m pole deflections. The optimal parameter space was further bounded by limiting pole stiffness (upper bound) in order to match peak shoulder forces expected of a rigid backpack carrying a similar load.

**Fig 8:**
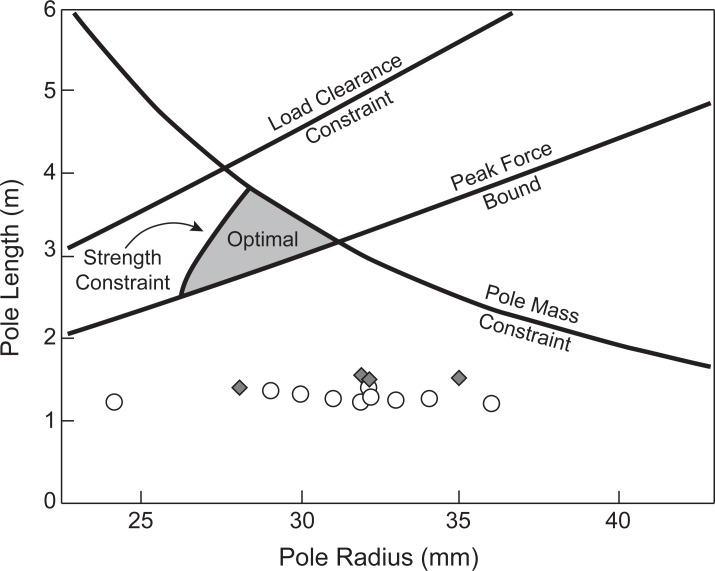
Measured pole parameters versus parameters of optimization model. Pole parameters (radius and length) are compared to the shoulder force optimization model developed by Potwar et al. [10]. Four constraints were used to determine a region of pole parameters that minimize forces felt at the shoulder. All 14 poles from the current study are also plotted (light circles are the FPs and dark diamonds are the LPs) for comparison.

In Fig 8, both the FPs (white circles) and LPs (grey diamonds) are plotted over the parameter space bounding the optimal range for pole design. This figure is a recreation of the model developed by Potwar et al. for a carrier walking at 1.34 ms^-1^ with Mao Zhu bamboo [note this species of bamboo (i.e. *Phyllostachys edulis*) is commonly found in northern regions of Vietnam [19] near the Thai Nguyen province where our poles were collected]. Although parameter optimizations were conducted for other conditions, this comparison was chosen simply because the optimal region is closest to the pole parameters measured in our study. Although all of the pole parameter combinations (pole length and outer radius) are clearly outside of the optimal range, there are a few reasons why this may be the case.

Specifically, the optimal parameter range assumes a pole with a semi-circular cross-sectional geometry, which is thicker (greater cross-sectional height) than the pole geometries observed in CT scans. As a result, applying our height measures to the Potwar et al. model results in an erroneous stiffness estimate. Still, the suggested optimal pole length is likely too far off for cross-sectional geometry to account for this discrepancy alone.

Although the optimal parameter space considers multiple constraints/bounds, there is perhaps an additional consideration left unaddressed: the effect of pole length on practicality and maneuverability. The current model predicts an optimal pole length of around 3 m and often more, (depending on bamboo species, cross-sectional properties, and walking speed). While this length may not pose too much difficulty for an individual working alone in a field, it would make loading and handling of the pole difficult in a crowded marketplace. It is possible that our poles were fabricated in part to facilitate maneuverability.

The model indicates optimal parameters that reduce forces distributed over the bearing surface of the shoulder. However, since our poles do not meet these optimality constraints, perhaps it is fair to conclude that they are manufactured to meet different design goals, or optimize a different aspect of the interaction between the individual and the tool. Here, we consider an alternative: the resonant oscillation of the pole-load system is tuned to the cadence of the carrier, to exploit an energetic benefit.

A thorough consideration of how pole properties influence locomotion energetics likely requires a rigorous model validated through empirical data. However, it may be useful to consider the general range of resonant frequencies, since reaction forces (felt by the carrier) increase with larger oscillations of the load and oscillations typically spike at, and around, resonant modes. For example, Castillo et al. [2] found that individuals received energetic benefits when they walked at a step frequency slightly above the resonant oscillations of the pole-load system. However, this is likely only feasible when the resonant frequencies are in the approximate range of a person’s preferred step frequency.

Typical preferred walking conditions include a step frequency range of approximately 1.5–2.0 Hz and a velocity of 1.0–1.5 ms^-1^ [20,21]. While this range of frequencies approximately coincides with the resonant frequencies of the LPs at larger load levels (see Fig 6), they are somewhat below the resonance curves for the FPs even at high loads (this difference is largely due to increased load stiffness resulting from the generally shorter lengths of these poles). Still, these comparisons are largely qualitative (i.e. non-rigorous) and do not take into consideration potential frequency responses associated with carrying rigid or oscillating loads.

For example, subtle increases of walking frequency tend to occur when a person carries a rigid load, although these changes are often insignificant [3,22]. At the same time, increases in walking speed are associated with increases in step frequency [21,23]. Therefore, the pole resonant frequencies may benefit the energetics of relatively fast walking, which may be appropriate for the increased pace of busy work on the farm or in the marketplace. In summary, if there *is* indeed an energetic benefit to walking with these poles, they would likely exist with heavier loads, 200 N (20 kg) per pole end or more (common load levels during farm work) and at relatively fast walking speeds. Regardless, future research would benefit from investigating more sophisticated models capable of predicting the motor behavior of locomotion when interacting with the flexible oscillations of different loads. However, such models should be thoroughly validated with rigorous empirical studies assessing locomotion of experienced users under natural conditions.

## Conclusions

A number of objectives were met by this study. We tested and assessed the mechanical properties of the four LPs (fabricated in a Vietnamese village according to traditional methods), which allowed us to describe basic dynamic behaviors inherent to their structure, material and design. Through this series of tests, we attained a set of fundamental parameters and properties. These included Young’s modulus of the bamboo, hysteresis and resilience of static loading/unloading, the rate of energy loss due to viscous damping occurring during free vibration, the second moment of area of the pole cross-sections and the resonant behaviors of the poles vibrating under load.

We applied a theoretical model using classical beam theory (of a cantilever beam with a partial tubular cross-section) to a mass-spring-damper system to predict the resonant behavior of differing loads. This model was experimentally validated for the four LPs. Finally, we used the theoretical model to determine the same set of mechanical and structural properties for the other 10 FPs.

These measurements provide a foundation for models evaluating the role of pole use and function by traditional cultures using this technology. Although Western cultures rely on a fixed load attachment such as a strapped backpack, this solution may be less effective and less energetically economical than interacting with the dynamic oscillations of a flexible bamboo pole. However, if the mechanisms of such interactions are to be determined, then the poles themselves must be thoroughly evaluated and understood. With the results presented here, a thorough and rigorous human locomotion model can now be used to investigate such interactions.

## Supporting information

S1 STL An STL file containing CT data of cross-sectional slices for Pole C. (STL)

S2 STL An STL file containing CT data of cross-sectional slices for Pole D. (STL)

S3 STL An STL file containing CT data of cross-sectional slices for Pole E. (STL)

S4 STL An STL file containing CT data of cross-sectional slices for Pole F. (STL)

S1 Text Raw outputs of inertial sensors for resonance testing on all poles. (ZIP)

S2 Text Digitized video tracking data for load deflection testing on all lab-tested poles. (ZIP)
